# The outcomes of acute myocardial infarction patients comorbidity with hypertension and hyperhomocysteinemia

**DOI:** 10.1038/s41598-021-02340-w

**Published:** 2021-11-25

**Authors:** Jumei Yan, Jiamin Zhou, Jun Huang, Hongyu Zhang, Zilin Deng, Yun Du

**Affiliations:** Department of Cardiology, The First Affiliated Hospital of Nanchang University, 17 Yongwaizheng St, Nanchang, 330006 Jiangxi China

**Keywords:** Cardiology, Medical research

## Abstract

This study investigated the outcomes and major adverse cardiovascular events (MACEs) incurred by acute myocardial infarction (AMI) patients comorbiding with hypertension and hyperhomocysteinemia (HHcy) during hospitalization and 1-year follow-up. 648 consecutive AMI patients were divided into four categories: (1) hypertension with Hcy ≥ 15 µmol/L; (2) hypertension with Hcy < 15 µmol/L; (3) no-hypertension with Hcy ≥ 15 µmol/L; (4) no-hypertension with Hcy < 15 µmol/L. Information taken from these case files included gender, past medical history, vital signs, laboratory examination, electrocardiogram, coronary angiography, cardiac ultrasound, and medicine treatment. The primary endpoints were duration of coronary care units (CCU) stay, duration of in-hospital stay, and MACEs during follow-up. Our data show that hypertension and HHcy have a synergistic effect in AMI patients, AMI comorbiding with hypertension and HHcy patients had more severe multi-coronary artery disease and more frequent non-culprit coronary lesions complete clogging, had a higher prevalence of pro-brain natriuretic peptide, and significant decreases in the left ventricular ejection fraction. These patients had significant increases in the duration of CCU stay and in-hospital stay, had significant increase in the rate of MACEs, had significant decreases in the survival rate during follow-up.

## Introduction

Cardiovascular diseases are the leading causes of mortality worldwide, and acute myocardial infarction (AMI) is one of the most fatal cardiovascular diseases. In the past years, traditional risk factors contributing to AMI had been recognized and treated. Because of the deep understanding of AMI and complications, the treatment effect and long-term survival rate of AMI patients are constantly improved. However, despite predictive strategies, a great number of AMI cases and outcomes remain unpredicted.

Homocysteine (Hcy) is a nonessential amino acid that is known as a marker of endothelial injury which is the starting point of atherosclerosis^1^. Elevated total Hcy was known to be able to increase oxidative stress, stimulate the proliferation of vascular smooth muscle cells, and thus increase the risk for atherosclerosis^2^. Hcy had been shown to be strongly associated with restenosis and major adverse cardiovascular events (MACEs) after coronary angioplasty, and with all-cause mortality^3,4^. Hypertension was an independent risk factor of carotid atherosclerotic plaques formation and rupture^5^. Previous studies had shown that hypertension and hyperhomocysteinemia (HHcy) could have a synergistic effect on the risk of cardio-cerebrovascular disease^6^.

Epidemiological studies demonstrated similar distributions of HHcy and hypertension, and both were related to an increased risk of cardiovascular events^7^. However, the outcomes of AMI patients with hypertension and HHcy are unclear. It will be worthwhile to determine if clinical outcomes are worse in AMI patients comorbidity with hypertension and HHcy. The purpose of this study is to investigate the clinical characteristics, length of in-hospital stay, and MACEs during hospitalization and 1-year follow-up in AMI patients with hypertension and HHcy.

## Results

### Baseline characteristics and outcomes of patients

The baseline clinical characteristics and outcomes of 648 enrolled patients were summarized in Table 1. The whole population were divided into four categories: (1) hypertension patients with Hcy ≥ 15 µmol/L (*n* = 202, 31.17%); (2) hypertension patients with Hcy < 15 µmol/L (*n* = 157, 24.23%); (3) no-hypertension patients with Hcy ≥ 15 µmol/L (*n* = 153, 23.61%); (4) no-hypertension patients with Hcy < 15 µmol/L (*n* = 136, 20.99%). There were no significant differences in gender, heart rate, diastolic blood pressure, smoking, diabetes mellitus, and impaired renal function. However, for hypertension patients, there was a higher prevalence of age (66.83 ± 12.09 vs. 61.32 ± 11.90, *P* < 0.001), past hypertension medical history (45.0% vs. 33.1%, *P* = 0.022), past dyslipidemia medical history (11.4% vs. 5.1%, *P* = 0.009), and systolic blood pressure (145.43 ± 20.00 vs. 139.72 ± 18.42, mmHg, *P* = 0.006) in the HHcy group.

**Table 1:** Comparison baseline characteristics of AMI patients in different groups.

	Hypertension		*P*	No-hypertension		*P*
	HCY ≥ 15 µmol/L n = 202	HCY < 15 µmol/L n = 157		HCY ≥ 15 µmol/L n = 153	HCY < 15 µmol/L n = 136	
**General conditions**						
Age (years)	66.83 ± 12.09	61.32 ± 11.90	0.000	62.84 ± 12.47	61.85 ± 11.86	0.488
Female	47 (23.3%)	50 (31.8%)	0.069	30 (19.6%)	20 (22.1%)	0.608
HR (bpm)	82.25 ± 19.68	79.54 ± 14.92	0.139	77.78 ± 16.97	78.87 ± 16.69	0.650
SBP (mmHg)	145.43 ± 20.00	139.72 ± 18.42	0.006	112.98 ± 17.14	114.53 ± 10.96	0.451
DBP (mmHg)	85.52 ± 12.31	85.22 ± 12.65	0.820	68.86 ± 10.46	71.93 ± 8.903	0.293
Smoking	46 (22.77%)	35 (22.29%)	0.894	32 (20.9%)	20 (14.7%)	0.170
**Past medical history**						
Hypertension	91 (45.0%)	52 (33.1%)	0.022			
Dyslipidemia	23 (11.4%)	8 (5.1%)	0.009	20 (13.1%)	11 (8.1%)	0.172
Diabetes mellitus	28 (13.9%)	13 (8.3%)	0.099	34 (22.2%)	11 (8.1%)	0.172
**Blood feature**						
TC (mmol/L)	4.31 ± 1.26	4.40 ± 1.51	0.550	4.22 ± 1.02	4.58 ± 1.24	0.243
TG (mmol/L)	1.72 ± 1.21	1.87 ± 2.32	0.450	1.07 ± 0.62	1.28 ± 0.79	0.152
HDL (mmol/L)	1.19 ± 0.82	1.19 ± 0.48	0.980	1.19 ± 0.85	1.18 ± 0.61	0.930
LDL (mmol/L)	2.70 ± 1.12	2.87 ± 1.07	0.140	2.70 ± 0.94	2.85 ± 1.22	0.322
Creatinine (mmol/L)	111.23 ± 54.29	78 ± 38.93	0.000	86.80 ± 30.79	69.15 ± 22.10	0.000
Uric acid (mmol/L)	361.29 ± 102.73	314.04 ± 96.27	0.000	319.86 ± 92.75	307.91 ± 85.06	0.264
D-dimer	0.70 ± 1.03	0.47 ± 1.16	0.066	1.67 ± 2.43	0.53 ± 0.78	0.001
FBG (mmol/L)	5.68 ± 1.67	5.92 ± 1.88	0.273	5.68 ± 1.67	5.72 ± 1.35	0.845
HCY	29.00 ± 10.37	10.87 ± 3.41	0.000	25.73 ± 17.32	11.80 ± 2.40	0.000
Pro-BNP (pg/mL)	628 (1311, 2125)	265 (680, 1324)	0.009	214 (818, 1973)	208 (710, 1232)	0.239
**Echocardiography**						
EF %	48.78 ± 9.18	53.20 ± 6.96	0.021	52.36 ± 7.92	54.25 ± 8.92	0.256
**ECG**						
STEM	141 (69.8%)	111 (70.7%)	0.853	109 (71.2%)	80 (58.8%)	0.027
NSTEMI	61 (30.2%)	46 (29.3%)	0.853	44 (28.7%)	56 (41.2%)	0.027
**Cardiac medication**						
Aspirin	192 (95.0%)	144 (91.7%)	0.201	143 (93.5%)	123 (90.4%)	0.908
Clopidogrel	194 (96.0%)	146 (93.0%)	0.201	143 (93.5%)	133 (97.8%)	0.076
Statin	192 (95.0%)	148 (94.3%)	0.743	142 (92.8%)	130 (95.6%)	0.316
Beta-blocker	109 (54.0%)	94 (59.9%)	0.262	74 (48.4%)	60 (44.1%)	0.470
ACEI/ARB	137 (67.8%)	97 (61.8%)	0.234	80 (52.3%)	77 (56.6%)	0.481
Diuretic drugs	38 (18.8%)	27 (17.2%)	0.694	22 (14.4%)	17 (12.5%)	0.641

Data are shown as mean ± standard deviation or n (%). Data in bold indicates *P*-values 0.05.

HR, heart rate; SBP, Systolic blood pressure; DBP, diastolic blood pressure; CABG, coronary artery bypass grafting; PCI, percutaneous coronary intervention; HDL, high density lipoprotein; LDL, low density lipoprotein; TC, total cholesterol; TG, triglyceride; UA, uric acid; FBG, fasting blood-glucose; EF, eject fraction; ECG, electrocardiograph; STEMI, ST segment elevation myocardial infarction, NSTEMI, NON-ST segment elevation myocardial infarction, ACEI, angiotensin converting enzyme inhibitor; ARB, angiotensin receptor blocker.

Biochemical variables at admission were shown in Table 1, there were no significant differences in total cholesterol (TC), triglyceride (TG), low-density lipoprotein cholesterol (LDL-C), high-density lipoprotein cholesterol (HDL-C), D-dimer, and fasting blood glucose (FBG). However, for hypertension patients, there was a higher prevalence of creatinine (111.23 ± 54.29 vs. 78 ± 38.93, mmol/L, *P* < 0.001), uric acid (361.29 ± 102.73 vs. 314.04 ± 96.27, mmol/L, *P* < 0.001), and pro-brain natriuretic peptide (pro-BNP) (628 (1311, 2125) vs. 265 (680, 1324), pg/mL, *P* = 0.009) in the HHcy patients. As shown in Table 1, whether in hypertension patients or in no-hypertension patients, the incidence of ST-segment elevated myocardial infarction (STEMI) (69.8% and 71.2%) was significant increase comparing with that of non-ST-segment elevated myocardial infarction (NSTEMI) (30.2% and 28.7%) in HHcy patients, but this was in contrast to the incidence in hypohomocysteine patients. For hypertension patients, there was significant decreases in the left ventricular ejection fraction (LVEF) (48.78 ± 9.18% vs. 53.20 ± 6.96%, *P* = 0.021) in the HHcy patients. In our study, no differences were observed in the use of aspirin, clopidogrel, beta blocker, statin, or Diuretic drugs among the four groups. However, the hypertensive patients more frequently received angiotensin converting enzyme inhibitor or angiotensin receptor blocker treatment than the no-hypertensive patients (65.19% vs. 54.32%).

### Angiographic and procedural characteristics

Angiographic and procedure characteristics at admission were shown in Table 2. For hypertension patients, coronary artery disease was more severe in the HHcy patients that presented with multi-vessel disease (61.4% vs.47.8%, *P* = 0.010) and more frequent non-culprit vessels chronic total occlusion (14.9% vs. 7.6%, *P* = 0.035), and intra-aortic balloon pump (IABP) was more frequently used in the HHcy patients (8.4% vs. 3.2%, *P* = 0.040). Comparing to patients with hypertension or HHcy alone, the coronary artery lesions of hypertension with HHcy patients were more located in the left main artery (10.4%). For no-hypertension patients, there was a trend of increased incidence of multi-vessel disease and non-culprit coronary lesions (NCCLs) chronic total occlusion in the HHcy patients, but these did not reach a significant value. There was no significant difference in the prevalence and extent of culprit vessels among the all four groups.

**Table 2:** Angiographic and procedural characteristics.

	Hypertension		*P*	No-hypertension		*P*
	HCY ≥ 15 µmol/L n = 202	HCY < 15 µmol/L n = 157		HCY ≥ 15 µmol/L n = 153	HCY < 15 µmol/L n = 136	
**Diseased vessels**						
LM	21 (10.4%)	14 (8.9%)	0.639	12 (7.8%)	8 (5.9%)	0.512
LAD	64 (31.7%)	40 (25.5%)	0.199	44 (28.8%)	32 (23.5%)	0.314
LCX	48 (23.8%)	40 (25.5%)	0.708	39 (25.5%)	24 (17.6%)	0.107
RCA	56 (27.7%)	37 (23.6%)	0.373	28 (18.3%)	21 (15.4%)	0.518
Multi-vessel disease	124 (61.4%)	75 (47.8%)	0.010	77 (50.3%)	54 (39.7%)	0.005
NCCLs clogging	30 (14.9%)	12 (7.6%)	0.035	15 (9.8%)	6 (4.4%)	0.419
IABP use	17 (8.4%)	5 (3.2%)	0.040	7 (4.6%)	2 (1.5%)	0.239

LM, left main; LAD, left anterior descending coronary artery; LCX, Left circumflex artery; RAD, right coronary artery; NCCLs, non-culprit coronary lesions; IABP, intra-aortic balloon pump.

### The primary endpoints and its components

First, the primary endpoints and its components during hospitalization were analysed. For hypertension patients, there were significant increase in the duration of coronary care units (CCU) stay (4.31 ± 2.61 vs. 3.32 ± 2.05, *P* < 0.001) and the duration of hospital stay (13.11 ± 4.95 vs. 11.94 ± 4.43, *P* = 0.020) in the HHcy patients; the rate of MACEs (defined as cardiovascular death, acute heart failure, and arrhythmia complications) (23.8% vs. 14.0%, *P* = 0.021) were also increase in the HHcy patients. When the components of MACEs were further investigated, we found that the cardiac death rate (7.9% vs. 3.8%, *P* = 0.108) and arrhythmia complications rate (11.9% vs. 6.4%, *P* = 0.077) increased in the HHcy patients (Table 3). For no-hypertension patients, there were a trend of increased such as the MACEs rate, the cardiovascular death rate, duration of CCU stay, and duration of hospital stay in the HHcy patients, but these did not reach a significant value (Table 3).

**Table 3:** The primary endpoint of in-hospital and 1-year follow-up outcomes.

	Hypertension		*P*	No-hypertension		*P*
	HCY ≥ 15 µmol/L	HCY < 15 µmol/L		HCY ≥ 15 µmol/L	HCY < 15 µmol/L	
**In-hospital events**						
Number of cases	202	157		153	136	
Cardiac death	16 (7.9%)	6 (3.8%)	0.108	5 (3.3%)	4 (2.9%)	1.000
Arrhythmia complications	24 (11.9%)	10 (6.4%)	0.077	12 (7.8%)	6 (4.4%)	0.228
Acute heart failure	27 (13.4%)	14 (8.9%)	0.189	16 (10.5%)	7 (5.1%)	0.096
MACEs	48 (23.8%)	22 (14.0%)	0.021	18 (11.8%)	12 (8.8%)	0.413
CCU stay	4.31 ± 2.61	3.32 ± 2.05	0.000	4.18 ± 2.76	3.88 ± 2.80	0.358
Hospital stay	13.11 ± 4.95	11.94 ± 4.43	0.020	11.41 ± 4.68	9.53 ± 3.96	0.108
**1-Year events**						
Number of cases	174	139		130	121	
Cardiac death	10 (5.7%)	4 (2.9%)	0.222	4 (3.1%)	0 (0%)	0.150
Repeat MI	8 (4.6%)	4 (2.9%)	0.431	4 (3.1%)	2 (1.7%)	0.746
Repeat PCI/CABG	15 (8.6%)	7 (5.0%)	0.218	12 (9.2%)	6 (5.0%)	0.190
MACEs	30 (17.2%)	12 (8.6%)	0.026	11 (8.5%)	7 (5.8%)	0.412

MACEs, major adverse cardiovascular events; CCU, critical care unit; AMI, myocardial infarction; CABG, coronary artery bypass grafting; PCI, percutaneous coronary intervention.

**Figure 1:**
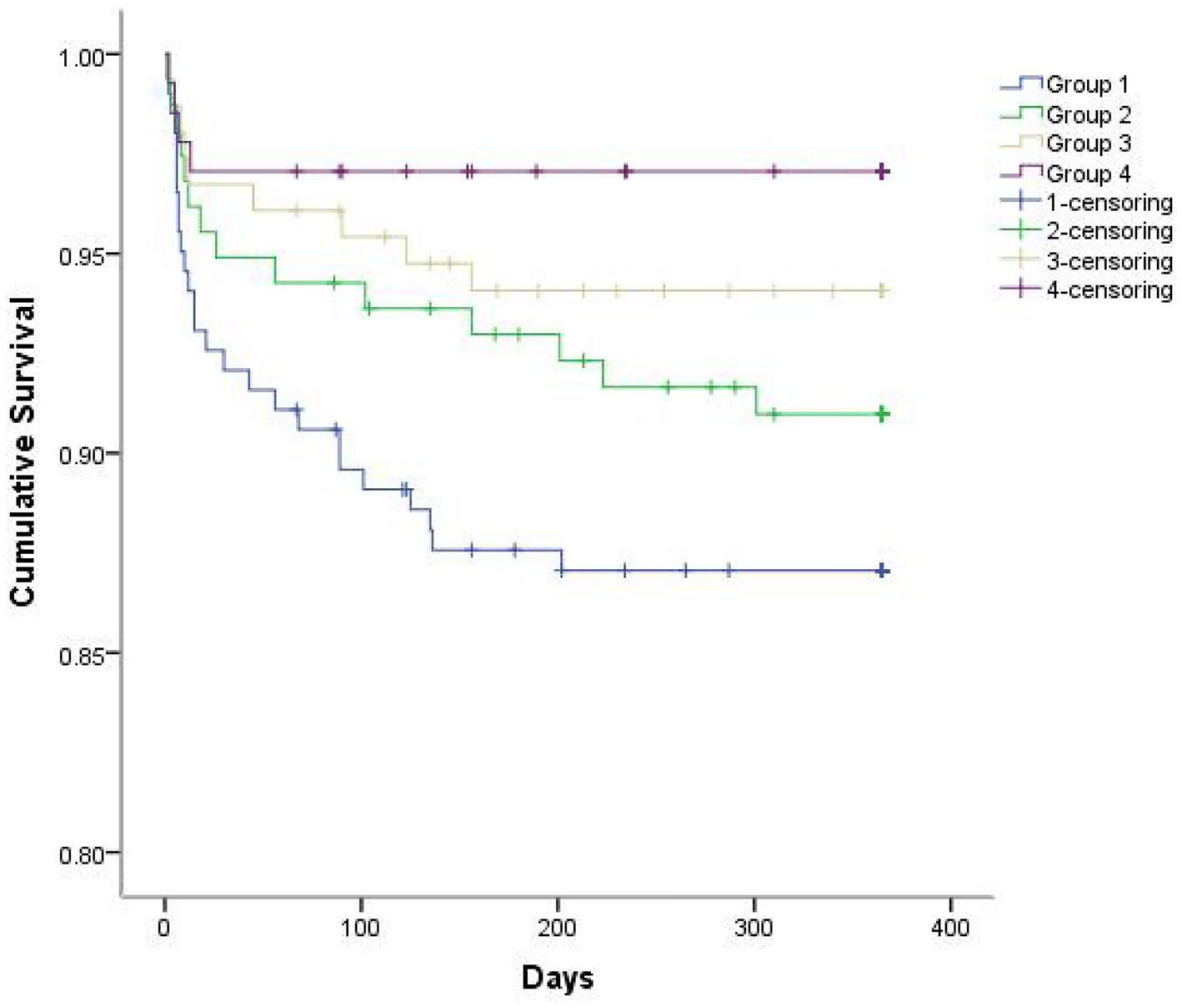
Kaplan–Meier survival curve of cumulative survival at 1-year follow-up for whole study population (log-rank test, *P* = 0.008). (group 1, hypertension with Hcy ≥ 15 µmol/L; group 2, hypertension with Hcy < 15 µmol/L; group 3, no-hypertension with Hcy ≥ 15 µmol/L; group 4, no-hypertension with Hcy < 15 µmol/L).

Then the primary endpoints and its components during 1-year follow-up were analysed. For hypertension patients, there were significant increase in the rate of MACEs (defined as cardiovascular death, repeat MI, and repeat PCI/CABG) (17.2% vs. 8.6%, *P* = 0.026) in the HHcy patients. When the componensts of MACEs were further investigated, we found that the cardiovascular death rate (5.7% vs. 2.9%, *P* = 0.222) increased in the HHcy patients, but there were only a trend of increased the incidence of repeat MI, and repeat revascularization, these did not reach a significant value (Table 3). For no-hypertension patients, there were a trend of increased the incidence of MACEs and cardiovascular death rate, but these did not reach a significant value (Table 3). Kaplan Meier analysis of cumulative survival at 1-year follow-up demonstrated that the significant survival down-regulated in hypertension comorbidity with HHcy group comparing with other three groups (Fig. 1).

The results of univariate and multivariate analysis using Cox proportional hazard model adjusting for other potential predictors of mortality are shown in Table 4. Univariate analysis hazard ratio (HR) and 95% confidence intervals (95% CI) determined that age ≥ 65 years (HR = 1.054, 95% CI 1.043–1.068, *P* = 0.019), female sex (HR = 0.671, 95% CI 0.644–0.896, *P* = 0.021), smoking (HR = 1.452, 95% CI 1.099–1.989, *P* = 0.013), dyslipidemia (HR = 1.765, 95% CI 1.592–1.989, *P* = 0.013), diabetes mellitus (HR = 1.205, 95% CI 1.060–1.371, *P* = 0.001), low LVEF (< 40%) (HR = 1.538, 95% CI 1.003–2.360, *P* = 0.001), and multivessel disease (HR = 2.038, 95% CI 1.603–2.660, *P* = 0.001) were all associated with a higher MACEs rate at 1-year follow-up in AMI patients. After adjusting for many covariates, age ≥ 65 years (HR = 0.745, 95% CI 0.654–0.884, *P* = 0.021), dyslipidemia (HR = 0.975, 95% CI 1.021–1.120, *P* = 0.034), diabetes mellitus (HR = 0.902, 95% CI 0.865–0.947, *P* = 0.022), and multi-vessel disease (HR = 1.721, 95% CI 1.468–2.054, *P* = 0.001) were independent predictors of 1-year MACEs. Also, AMI patients with hypertension or HHcy alone, especially AMI patients with hypertension and HHcy, significantly increased the risk of MACEs at 1-year follow-up in AMI patients.

**Table 4:** Univariate and multivariate analysis for independent predictors of 1-year MACEs.

	Unadjusted HR	95% CI		*P* valve	Adjusted HR	95% CI		*P* valve
		Lower	Upper			Lower	Upper	
Age (≥ 65 years)	1.054	1.043	1.068	0.019	0.745	0.654	0.884	0.021
Female sex	0.671	0.644	0.896	0.021	0.578	0.563	1.428	0.725
Smoking	1.452	1.099	1.790	0.024	1.058	0.845	1.217	0.543
Dyslipidemia	1.765	1.592	1.989	0.013	0.975	1.021	1.120	0.034
Diabetes mellitus	1.205	1.060	1.371	< 0.001	0.902	0.865	0.974	0.022
Low LVEF (< 40%)	1.538	1.003	2.360	< 0.001	0.803	0.667	0.963	0.063
STEMI	1.113	0.648	1.183	0.654	0.873	0.567	1.263	0.587
Multivessel disease	2.038	1.603	2.660	< 0.001	1.721	1.468	2.054	< 0.001
**No HNT no HHCY**								
HNT no HHCY	1.616	1.253	2.083	0.063	1.365	1.154	1.896	0.045
HCY no HNT	1.809	1.458	2.245	< 0.001	1.532	1.239	2.032	0.012
HNT and HHCY	2.014	2.464	2.142	< 0.001	1.765	1.984	2.012	< 0.001

MACE, major adverse cardiac events; HR, hazard ratio; CI, confidence interval; LVEF, left ventricular ejection fraction; PCI, percutaneous coronary intervention; STEMI, ST-segment elevation myocardial infarction; HNT, hypertension; HHCY, high homocysteine.

## Discussion

To the best of our knowledge, the present study is the first to report the baseline characteristics and outcomes of AMI patients comorbidity with hypertension and HHcy. Our novel major findings are summarized as follows: (1) AMI patients with hypertension and HHcy have more severe multi-coronary artery disease and more frequent NCCLs chronic total occlusion; (2) AMI patients with hypertension and HHcy have a higher prevalence of pro-BNP and significant decreases in LVEF; (3) AMI patients with hypertension and HHcy have significant increase in the duration of CCU stay and in-hospital stay; (4) AMI patients with hypertension and HHcy have significant increase in the rate of MACEs during hospitalization and 1-year follow-up.

Hcy is a type of amino acid that contains sulfur and is produced by methionine metabolism. HHcy was believed to promote atherogenesis and atherothrombosis through several mechanisms^2^. Hcy metabolism generated reactive oxygen species that could directly injure the endothelium. Hcy had been shown to inhibit nitric oxide synthase, leading to endothelial dysfunction. 10% of general population coronary artery disease (CAD) risk was attributed to Hcy^8^. The elevated Hcy concentration was closely related to the mortality and morbidity of CAD^9^. The risk of coronary artery disease would be increased by 60% for men and 80% for women with an elevation of 5 µmol/L in plasma Hcy^8^. HHcy was not only associated with CAD but also with AMI. In AMI patients, elevated Hcy level was associated with a higher risk of coronary events and death, independently of other risk factors^10^. An elevated plasma Hcy level was an independent predictor of the incidences of heart failure, cardiac rupture, death, and the total 30-day cardiovascular events in AMI patients^11^. Plasma Hcy concentration was an independent predictor for long-term mortality and MACE in ACS octogenarians after controlling conventional cardiovascular risk factors^12^. Culprit lesions, the treatment of which was the primary goal of PCI, had been found to be responsible for AMI, while NCCLs were considered to be innocent^13^. Hcy was an independent risk factor for NCCLs progression after 12 months of follow-up in elderly patients with ACS who had undergone PCI^14^. In the present study, we found that for hypertension patients, coronary artery atherosclerosis was more severe that presented with multi-vessel disease (61.4% vs. 47.8%, *P* = 0.010), more frequent in non-culprit vessels chronic total occlusion (14.9% vs. 7.6%, P = 0.035), and significant decreases in LVEF (48.78 ± 9.18% vs. 53.20 ± 6.96%, *P* = 0.021) in the HHcy patients. Because of these disease feature, there were significant increase in the CCU stay (4.31 ± 2.61 vs. 3.32 ± 2.05, *P* < 0.001) and hospitalization stay (13.11 ± 4.95 vs. 11.94 ± 4.43, *P* = 0.020), the rates of MACEs during hospitalization or during 1-year follow-up also increased in the HHcy patients. Among the no-hypertension patients, we also found that coronary artery disease was more severe that presented with multi-vessel disease (50.3% vs. 39.7%, *P* = 0.005) in the HHcy patients. Our data was similar with previous study, which suggested the HHcy could predict the risk and outcomes of patients with AMI.

Hypertension was one of the main risk factors for atherosclerosis development. Some pathophysiologic mechanisms involved in the genesis of hypertension (such as endothelial dysfunction, insulin resistance, and diabetes) were also the risk factors for CAD^15^. Hypertension was shown to be associated with an increased rate of adverse outcomes after AMI such as heart failure and cardiovascular death^16^. HHcy had been linked to hypertension for the past years, elevated Hcy levels had been consistently reported in hypertensive patients. Previous studies found that hypertension and HHcy had shown a multiplicative effect, increased the risk of stroke^17^ and recurrent ischemic stroke^18^. Hypertension and HHcy should be the major intervention measures to decrease the incidence of carotid atherosclerotic plaques as well as the stroke^19^. Prevalence of events increased with increasing plasma Hcy levels suggesting a contribution of Hcy to cerebro-cardiovascular diseases in hypertensive patients^20^. HHcy were associated with impaired endothelial dependent vasodilatation in hypertension, Hcy and endothelium dysfunction might promote thrombogenesis and atherogenesis, leading to adverse cardiovascular events^21,22^. It had been well known that pathophysiologic mechanism of cerebro-cardiovascular atherosclerosis was identical. Then we supposed that hypertension comorbidity with HHcy might have multiplicative effect in the outcomes of AMI patients. In the present study, our data were similar with previous study. We found that for HHcy patients, coronary artery disease was more severe that presented with multi-vessel disease (61.4% vs. 50.3%), more frequent non-culprit vessels chronic total occlusion (14.9% vs. 9.8%), and significant increases in Pro-BNP (628 (1311, 2125) vs. 265 (680, 1324) pg/mL) in the hypertension patients. We also found that the rates of MACEs during hospitalization or during 1-year follow-up also increased in the hypertension patients when compared with no-hypertension patients. Kaplan–Meier survival analysis at 1-year follow-up demonstrated the significant down-regulation in hypertension with HHcy group compared with other three groups.

In conclusion, the results of this study could have some relevant implications for identification the outcomes and management of AMI patients comorbidity with hypertension and HHcy. Comparing to AMI patients with hypertension or HHcy alone, AMI patients comorbidity with hypertension and HHcy significantly increased the risk of MACEs at in-hospital and1-year follow-up, and decreased the survival rate at 1-year follow-up.

## Methods

### Study population and inclusion criteria

This study was a single center retrospective study based on the medical records of enrolled patients, which comprised 736 consecutive AMI patients, second-generation drug-eluting stent implantation in all patients, which were defined as NSTEMI or STEMI^23^, and who were admitted to our hospital from June 2018 to December 2020. Among them, 41 patients who were missing Hcy values were excluded. 47 patients with incomplete clinical data, hypothyroidism, and those who took medicine that could potentially affected the Hcy metabolism (such as folic acid, vitamins, and carbamazepine) were excluded. 648 patients were finally enrolled in this research. Information taken from these case files included gender, age, discharge diagnosis, history of smoking, hypertension, diabetes mellitus, dyslipidemia, Hcy, vital signs, laboratory examination, electrocardiogram, coronary arteriography, cardiac ultrasound, complications, and medicine treatment. The primary endpoints of this study were duration of CCU stay, duration of hospital stay, and MACEs defined as cardiovascular death, acute heart failure, arrhythmia, myocardial infarction (MI), and repeat revascularization (repeat percutaneous coronary intervention or coronary artery bypass grafting). Follow-up was obtained in every patient that survived to discharge by reviewing the medical records and / or by telephone interview with the patient or family members at 1-year after admission.

The study complied with the declaration of Helsinki for investigation in human beings and was approved by the Ethical Committee of The First Affiliated Hospital of Nanchang University. As the study obtained relevant information from the previous medical records, the Ethical Committee agreed that the researchers did not need to obtain the written consent of the relevant patients.

### Definitions

Hypertension was defined as blood pressureure ≥ 140/90 mmHg or the use of antihypertensive medications. Diabetes mellitus was defined as fasting plasma glucose ≥ 6.1 mmol/L and postprandial glucose ≥ 7.8 mmol/L, and/or diagnosed diabetes mellitus receiving treatment. Dyslipidemia was defined as current treatment with cholesterol-lowering medications or TC value of > 5.72 mmol/L and/or TG value of > 1.7 mmol/L and/or LDL-C value of > 3.12 mmol/L and/or HDL-C value of < 1.0 mmol/L. HHcy was defined as Hcy ≥ 15 mmol/L. Serum samples were measured in the first 48 h after admission from peripheral venous blood samples. Patients that had smoked during the previous 6 months were classified as smokers and were self-reported. Arrhythmia included atrial fibrillation, ventricular premature beat, ventricular tachycardia, and atrioventricular block. AMI was diagnosed if the patient fulfilled 2 of the following 3 standards: chest pain continuing for > 30 min; dynamic alteration of ST-T appears in the electrocardiogram (ECG); and dynamic increases in troponin I or creatine kinase MB greater than three times the upper limit of normal value. Coronary artery stenosis was diagnosed based on the presence of > 50% lumen obstruction of at least one of three major coronary arteries.

### Statistical analysis

All analyses were conducted using the statistical software SPSS statistics 20 (IBM Corp, Armonk, NY, USA), and *P* < 0.05 was considered to be statistically significant. Continuous variables were expressed as mean ± standard deviation or median (with inter-quartile range), and categorical variables as numbers and percentages. Comparisons between quartiles were made by analysis of variance test for continuous variables and the Pearson chi-square test for categorical variables. Survival analysis was performed by applying the Kaplan–Meier method and log-rank test. Multivariate COX regression was used to determine the main risk factors for MACEs in follow-up. Multivariate COX regression analysis adopted the entry method, and *P* < 0.05 (two-sided) was considered as the difference was statistically significant.
